# Exploitation of Precision Medicine Trials Data: Examples of Long Responders From the SHIVA01 Trial

**DOI:** 10.1200/PO.18.00048

**Published:** 2018-10-19

**Authors:** Clémence Basse, Claire Morel, Céline Callens, Gaëlle Pierron, Vincent Servois, Anne Vincent-Salomon, Aude Jobard, Marie Alt, Francesco Ricci, Delphine Loirat, Marie-Paule Sablin, Marie Bretagne, Mathilde Saint-Ghislain, Ségolène Hescot, Anthony Gonçalves, Olivier Tredan, Coraline Dubot, Céline Gavoille, Jean-Pierre Delord, Mario Campone, Nicolas Isambert, Lisa Belin, Ivan Bieche, Maud Kamal, Christophe Le Tourneau

**Affiliations:** **Clémence Basse**, **Claire Morel**, **Céline Callens**, **Gaëlle Pierron**, **Vincent Servois**, **Anne Vincent-Salomon**, **Aude Jobard**, **Marie Alt**, **Francesco Ricci**, **Delphine Loirat**, **Marie-Paule Sablin**, **Marie Bretagne**, **Mathilde Saint-Ghislain**, **Ségolène Hescot**, **Coraline Dubot**, **Ivan Bieche**, **Maud Kamal**, and **Christophe Le Tourneau**, Institut Curie, Paris; **Clémence Basse**, **Claire Morel**, **Aude Jobard**, **Marie Alt**, **Francesco Ricci**, **Delphine Loirat**, **Marie-Paule Sablin**, **Marie Bretagne**, **Mathilde Saint-Ghislain**, **Ségolène Hescot**, **Coraline Dubot**, **Lisa Belin**, **Maud Kamal**, and **Christophe Le Tourneau**, Institut Curie; **Christophe Le Tourneau**, Institut National de la Santé et de la Recherche Médicale U900 Research Unit, Saint-Cloud; **Anthony Gonçalves**, Institut Paoli-Calmettes, Marseille; **Olivier Tredan**, Centre Léon Bérard, Lyon; **Céline Gavoille**, Centre Alexis Vautrin, Nancy; **Jean-Pierre Delord**, Institut Claudius Régaud, Toulouse; **Mario Campone**, Centre René Gauducheau, Nantes; **Nicolas Isambert**, Centre Georges-François Leclerc, Dijon; and **Christophe Le Tourneau**, Versailles-Saint-Quentin-en-Yvelines University, Montigny-le-Bretonneux, France.

## Abstract

**Purpose:**

Precision medicine trials constitute a precious source of molecular data with prospective clinical annotations allowing the exploration of patients’ subpopulations according to specific clinical or biological questions. Using the SHIVA01—the first randomized trial comparing molecularly targeted therapy on the basis of tumor molecular profiling versus conventional chemotherapy in metastatic cancer patients who failed standard of care therapy—annotated database, we report cases of patients treated in the trial with targeted therapy who experienced an objective response or prolonged disease stabilization in light of patients’ molecular alterations.

**Patients and Methods:**

We selected all patients included in SHIVA01 treated with a molecularly targeted agent (MTA) who experienced an objective response or disease stabilization that lasted longer than 6 months according to Response Evaluation Criteria in Solid Tumors version 1.1.

**Results:**

Among the 170 patients who received MTAs in the SHIVA01 trial, 15 patients (9%) experienced an objective response (n = 3) or disease stabilization that lasted longer than 6 months (n = 12). The most frequent histologic subtypes were breast cancer (27%) and cervical cancer (20%). Six patients, including three patients with breast cancer, were treated with abiraterone on the basis of androgen receptor protein overexpression. Five patients were treated with everolimus on the basis of a *PTEN* heterozygous deletion with loss of protein expression, *PIK3CA* mutation, or both alterations. The remaining four patients were treated with tamoxifen, erlotinib, imatinib, and vemurafenib on the basis of progesterone receptor expression, *EGFR* amplification, *KIT* mutation, and *BRAF* mutation, respectively. *TP53* mutations were absent in responder patients.

**Conclusion:**

Analysis of patients who experienced objective responses or disease stabilization that lasted longer than 6 months allowed the identification of potential biomarkers of sensitivity and resistance to MTAs.

## INTRODUCTION

Some molecularly targeted agents (MTAs) have been demonstrated to dramatically improve the outcome of patients whose tumors harbor a matching molecular alteration.^1^ We know from The Cancer Genome Atlas data that most of the druggable molecular alterations, including gene mutations and gene copy number alterations, exist across various tumor types, although their prevalence and functional significance may vary.^2^ On the basis of the latter observation, a key question has emerged: Should patients with cancer be treated according to their molecular profile in a histology-agnostic way instead of by tumor type and histology, at least in the metastatic setting? Whereas nonrandomized and retrospective studies have suggested the histology-agnostic approach might be valid,^3-5^ the SHIVA01 trial—the first randomized precision medicine trial—did not demonstrate a significant difference in progression-free survival (PFS) between matched targeted therapy and conventional treatment in patients who eventually experienced progression after standard-of-care therapy.^6^ Some patients who were treated in the SHIVA01 trial, however, seemed to benefit from targeted therapy. Among patients who crossed over in SHIVA01, 37% of patients had a ratio of PFS on MTA to PFS on last treatment that exceeded 1.3.^7^ Although PFS ratio is not a validated end point, this result compared favorably with results obtained in the von Hoff study and in MOSCATO01.^3,5^

Precision medicine trials are a precious source of molecular data with prospective clinical annotations allowing the exploration of patients’ subpopulations according to specific clinical or biologic questions. Using the SHIVA01 annotated database, we report here cases of patients who were treated with targeted therapy who experienced an objective response or prolonged disease stabilization and discuss them in light of their molecular alterations and in the context of available preclinical and clinical literature.

## PATIENTS AND METHODS

The SHIVA01 trial was an open-label, randomized, controlled, phase II trial run by the Institut Curie in eight French cancer centers, the global results of which have been published.^6^ The objective of SHIVA01 was to assess the efficacy of marketed MTAs given outside their indications on the basis of tumor molecular profiling of a metastatic site as compared to conventional treatment at physician’s choice in patients with any kind of cancer who eventually progressed after standard of care therapy. All patients who were enrolled in SHIVA01 previously received the standard treatment approved for their indication, including MTAs, and had an Eastern Cooperative Oncology Group performance status of 0 or 1.^8^

Within the SHIVA01 trial, MTAs were administered according to a prespecified treatment algorithm and each MTA was administered according to a matched molecular biomarker (Data Supplement). The duration of the last therapy was not documented in the SHIVA01 clinical trial.^8^ Crossover was possible at disease progression in both treatment groups. Tumor evaluations were performed every 2 months until disease progression. No difference in PFS between the two arms was observed, which was the primary end point of the trial. Techniques used included next-generation sequencing (AmpliSeq cancer panel on an Ion Torrent/PGM system; Thermo Fisher Scientific, Waltham, MA) for detecting mutations, Cytoscan HD (Affymetrix, Santa Clara, CA) for gene copy number alterations, and immunohistochemistry for hormone receptor protein expression assessment. Next-generation sequencing analysis was standardized among the different wet platforms for the SHIVA01 trial, and the bioinformatics analyses were centralized at Institut Curie.^6^ Genetic alterations leading to cancer were recalled, including the abnormal activation of oncogenes (gain of function) and inactivation of tumor suppressor genes (loss of function).^8^

Patients who were treated with MTAs in the SHIVA01 trial at random assignment or at crossover and who experienced an objective response or disease stabilization that lasted longer than 6 months according to Response Evaluation Criteria in Solid Tumors (RECIST) version 1.1 were selected.

We extracted the following data from the SHIVA01 trial database: patient characteristics, tumor type, molecular alterations, MTAs received, and clinical outcomes, including best objective response and PFS. We also recorded the total number of patients in SHIVA01 with the same tumor type treated with the same drug on the basis of the same molecular alteration.

We assessed relationships between genomic alteration and clinical response using Fisher’s exact test. Welch’s two-sample *t* test was used to assess differences in molecular alterations between responders and nonresponders. Differences between two populations were considered significant at CIs greater than 95% (*P* < .05).

## RESULTS

### Patient Characteristics

In the SHIVA01 trial, 170 patients were treated with MTAs, including 100 patients at random assignment and 70 at crossover.^6-9^ Fifteen (9%) of these 170 patients met our selection criteria, including three patients with an objective response (2%) and 12 patients (7%) with disease stabilization that lasted longer than 6 months. Two of the 15 patients who received a median of two previous lines of therapy (range, 1 to 13) received the MTA at crossover. The most frequent histologic subtypes were breast (n = 4; 27%) and cervical cancer (n = 3; 20%) among 10 different tumor types (Table 1).

**Table 1.**
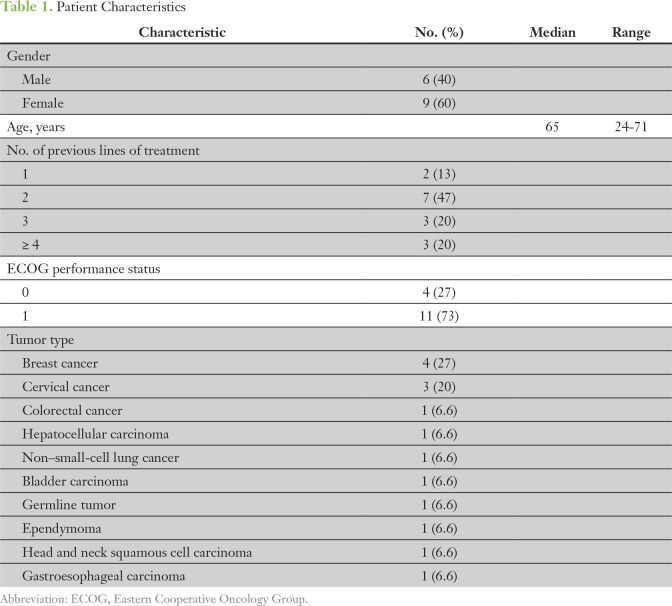
Patient Characteristics

### Molecular Profiles

Among the 15 patients who achieved an objective response or disease stabilization that lasted longer than 6 months, seven patients (47%) had a molecular alteration that involved the hormone receptor pathway (Table 2). Six patients who were treated with abiraterone had androgen receptor (AR) protein expression that ranged from 40% to 100%, including three patients with breast cancer, one patient with hepatocellular carcinoma, and one patient with bladder cancer (Table 2). The remaining patient with gastroesophageal cancer and a 30% progesterone receptor expression was treated with tamoxifen.

**Table 2.**
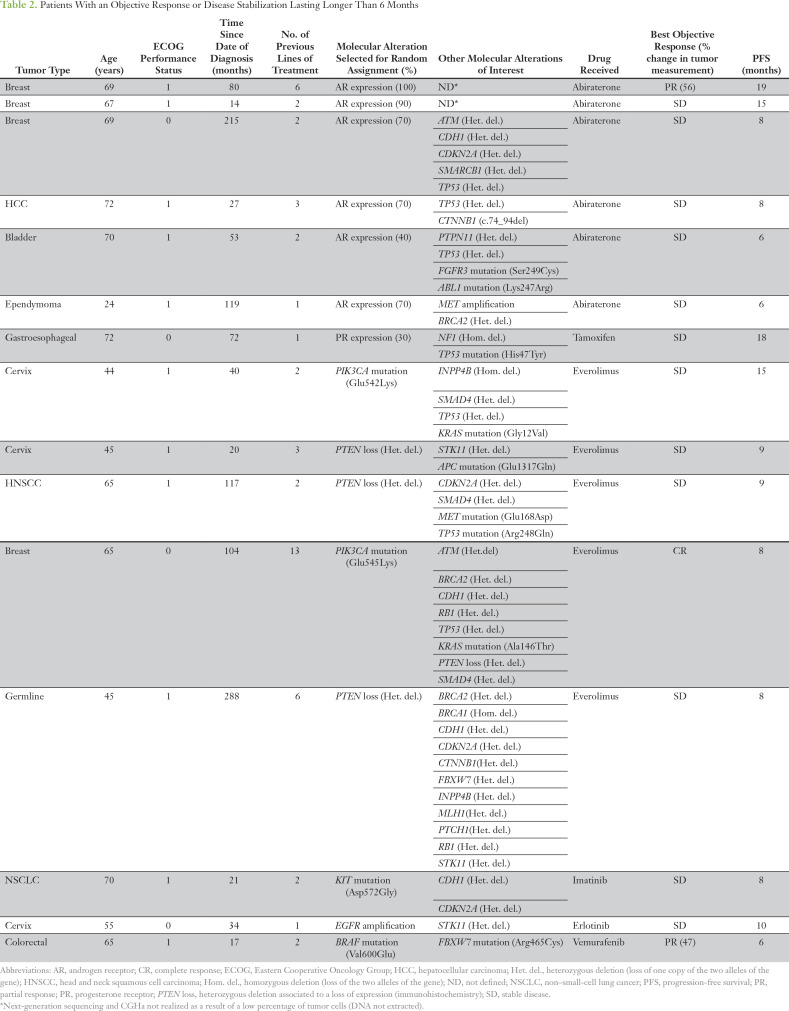
Patients With an Objective Response or Disease Stabilization Lasting Longer Than 6 Months

Five patients (33%) who were treated with everolimus had a molecular alteration involving the phosphatidylinositol 3-kinase (PI3K)/AKT/mammalian target of rapamycin (mTOR) pathway. Among these patients, three patients—cervical cancer, head and neck, and germline cancer—had a phosphatase and tensin homolog (*PTEN*) heterozygous deletion associated with a loss of protein expression. The two remaining patients consisted of one patient with cervical cancer with a *PIK3CA* mutation (Glu542Lys) and another with breast cancer with both *PTEN* loss and *PIK3CA* mutation (Glu545Lys). Four of these five patients—two with cervical cancer, one with breast cancer, and one with germline cancer—had at least two molecular alterations involving the PI3K/AKT/mTOR pathway, including *STK11* and *INPP4B* (Table 2).

Three patients were treated with imatinib, erlotinib, and vemurafenib on the basis of an Asp572Gly *KIT* mutation (lung cancer), an *EGFR* amplification (cervical cancer), and a Val600Glu *BRAF* mutation (colorectal cancer), respectively (Table 2).

### Comparative Analysis With Patients With No Objective Response or Disease Stabilization That Lasted Longer Than 6 Months

Three cohorts of patients from the SHIVA01 trial with objective response or disease stabilization that lasted longer than 6 months had more than a single patient with the same tumor type and were treated with the same MTA matching an alteration in a specific signaling pathway. In total, 22 patients with breast cancer were treated with abiraterone, 12 patients with breast cancer with everolimus, and 10 patients with cervical cancer with everolimus (Table 3). All other cohorts were single-patient cohorts. Within these three larger cohorts, the proportion of patients with an objective response or disease stabilization that lasted longer than 6 months varied from 8% to 20% (Table 3). Taken together, 15 (28%) of the 53 patients in these cohorts achieved an objective response or disease stabilization that lasted longer than 6 months (Table 3).

**Table 3.**
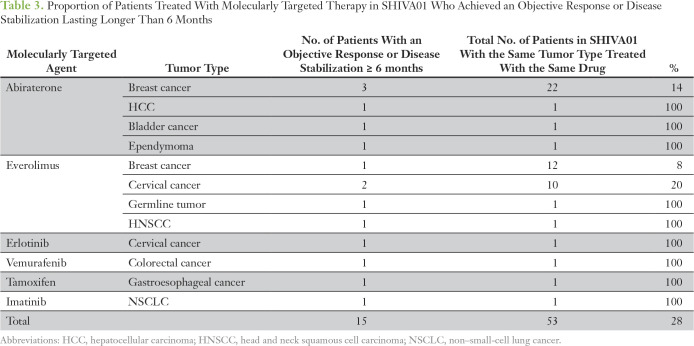
Proportion of Patients Treated With Molecularly Targeted Therapy in SHIVA01 Who Achieved an Objective Response or Disease Stabilization Lasting Longer Than 6 Months

#### Focus on patients treated with everolimus.

Theonly patient with breast cancer treated with everolimus who experienced a complete response had a *PIK3CA* mutation (Glu545Lys), as well as a *KRAS* mutation (Ala146Thr), associated with heterozygous deletions in the *ATM, BRCA2, CDH1, PTEN, RB1, SMAD4*, and *TP53* genes (Table 2 and Data Supplement). Of the 11 nonresponder patients with breast cancer, seven received everolimus on the basis of a *PIK3CA* mutation (3× His1047Arg, 2× Glu545Lys, and 2× Glu542Lys) and four received everolimus on the basis of *PTEN* inactivation. One patient had a *KRAS* mutation (Gly12Val), and five patients had a *TP53* mutation (Data Supplement).

The two patients with cervical cancer treated with everolimus who achieved disease stabilization that lasted longer than 6 months had either a *PIK3CA* mutation (Glu542Lys) or a *PTEN* heterozygous deletion with a loss of protein expression (Table 2 and Data Supplement). Among the eight nonresponder patients with cervical cancer, four received everolimus on the basis of *PIK3CA* mutation (Glu545Lys), two on the basis of *PTEN* inactivation, and two on the basis of *AKT1* mutation (Glu17Lys). Multiple coexisting molecular alterations were identified in these eight nonresponder patients, including 34 heterozygous deletions in tumor suppressor genes (Data Supplement). In total, among the three patients who achieved an objective response or disease stabilization that lasted longer than 6 months, all had a double alteration affecting the PI3K/AKT/mTOR pathway. Among the nonresponder patients with breast and cervical cancer treated with everolimus, only nine (47%) of the 19 patients had a double alteration in this pathway (*P* = .22).

#### Focus on patients treated with abiraterone.

The three patients with breast cancer treated with abiraterone who achieved an objective response or disease stabilization that lasted longer than 6 months had at least a 70% AR expression; however, six nonresponder patients had also an AR expression that exceeded 70%, among whom only one patient had no other molecular alteration (Data Supplement).

#### Impact of TP53 alteration in resistance to treatment.

Nonresponder patients with breast and cervical cancer treated with abiraterone or everolimus had more alterations in *TP53.* None of the four patients who experienced an objective response or disease stabilization that lasted longer than 6 months and for whom a complete molecular profile was available had a mutation in the *TP53* gene compared with 10 (31%) of 32 nonresponders with a complete molecular profile (*P* = .47; Data Supplement).

#### Impact of molecular alteration burden in resistance to treatment.

A median of five molecular alterations (range, three to eight) were detected in three patients treated with everolimus who experienced an objective response or disease stabilization that lasted longer than 6 months compared with a median of six alterations (range, two to 21) in 19 nonresponder patients. The number of alterations per responder versus nonresponder patients is illustrated in the Data Supplement. A median of three molecular alterations (range, one to eight) were found in three patients treated with abiraterone who experienced an objective response or disease stabilization that lasted longer than 6 months compared with a median of five alterations (range, one to 17) in 19 nonresponder patients. The number of alterations per responder versus nonresponder patients is illustrated in the Data Supplement. The total number of molecular alterations was not significantly associated with resistance to treatment (*P* = .41; Data Supplement).

## DISCUSSION

Using the SHIVA01 trial’s annotated database, we here report 15 patients (9%) who experienced an objective response or prolonged disease stabilization among the 170 patients with any kind of cancer treated with an MTA outside its indication in the SHIVA01 trial. These 15 patients represented 28% of the 53 patients in SHIVA01 with the same tumor type treated with the same MTA. *TP53* mutations were absent in responder patients. The total number of molecular alterations was not significantly associated with resistance to treatment, which suggests that other mechanisms that require additional investigation are involved.

Patients experiencing an objective response or a disease stabilization that lasted longer than 6 months were less heavily pretreated than the entire patient population that was included in the SHIVA01 trial.^6^ This result is in agreement with several reports in the literature that suggest that the sooner MTAs are administered in the course of the disease, the higher the efficacy.^1,10^

In the two largest cohorts in the SHIVA01 trial treated with everolimus or abiraterone, patients who experienced an objective response or disease stabilization that lasted longer than 6 months had fewer molecular alterations than nonresponder patients. Kurzrock and colleagues used a score, called Matching Score, that was calculated by dividing the number of MTAs administered by the number of druggable molecular alterations. Efficacy of MTAs administered in a manner similar to that of SHIVA01 correlated with the Matching Score.^11,12^

In agreement with a recent report, three of 22 patients with breast cancer expressing AR and treated with abiraterone experienced disease stabilization that lasted longer than 6 months.^13^ One patient with hepatocellular carcinoma, one with bladder carcinoma, and one with ependymoma treated with abiraterone in SHIVA01 experienced disease stabilization that lasted longer than 6 months; however, no evidence of efficacy of antiandrogens in these cancer types has been reported in the literature.^14-17^

One patient with gastroesophageal carcinoma treated with tamoxifen on the basis of progesterone receptor expression had an 18-month disease stabilization in the SHIVA01 trial. Antitumor activity of tamoxifen in hormone receptors expressing gastric cancer cells has been reported.^14^ To date, no clinical trials have been reported, to our knowledge, evaluating this strategy in the clinic.

All three patients treated with everolimus who achieved an objective response or disease stabilization that lasted longer than 6 months had a double alteration affecting the PI3K/AKT/mTOR pathway, whereas less than one half of nonresponder patients with breast and cervical cancer had a double alteration involving that pathway. One patient with recurrent head and neck squamous cell carcinoma harboring a *PTEN* loss experienced a 9-month disease stabilization with everolimus in SHIVA01. In a phase II trial that involved seven patients with recurrent and/or metastatic head and neck squamous cell carcinoma treated with everolimus without any molecular selection, a median PFS of 1.5 months was reported with no objective response.^15^ One patient with breast cancer (8%) with a *PTEN* loss and an activating PIK3CA mutation experienced an objective response that lasted 8 months in SHIVA01. Thirty-five percent of patients with breast cancer with an alteration in the PI3K/AKT/mTOR pathway who were treated with various therapies that inhibited the PI3K/AKT/mTOR pathway in the SAFIR01 study had an objective response or PFS that lasted longer than 16 weeks.^16^ The difference in terms of efficacy observed in SAFIR01 might be related to the 16-week threshold used for prolonged disease stabilization—instead of the 6 months in our study—and that various drugs used sometimes in combination, including direct PI3K inhibitors in SAFIR01. One patient with a germ-cell tumor was treated with everolimus in our study on the basis of a *PTEN* loss. In a phase II trial that evaluated everolimus in unselected patients with refractory testicular germ-cell tumors, no objective response was observed with a PFS rate at 3 months of 40%.^18^ Finally, three patients in SHIVA01 seemed to benefit from MTAs that targeted epidermal growth factor receptor, *KIT*, and BRAF mutations, which are clinically validated targets in other tumor types.^19-22^ Of interest, the patient with *BRAF* V600E–mutated colorectal cancer experienced an unusual response to vemurafenib. Partial responses to vemurafenib in patients with *BRAF* V600E–mutated colorectal cancer have previously been reported in the literature.^23^ The absence of *TP53* mutations and PI3K pathway alterations in these patients may explain the partial response to vemurafenib,^24^ although this remains speculative.

In conclusion, the design of the SHIVA01 trial has several limitations. First, only marketed drugs used outside of their indications were included in the treatment algorithm and were mainly administered as single MTA. It is now clear, for example, that everolimus was not the best drug to target *PI3KCA* mutations. Second, the treatment algorithm was unidimensional and did not account for resistance mechanisms.^9^ Third, heavily pretreated patients were included in the trial, which reduced the likelihood that MTAs might be effective. Despite these caveats, SHIVA01 allowed for the integration of clinically annotated molecular data that were used to analyze patients with unusual responses to MTAs.^6^

The exploitation of clinically annotated molecular data from precision medicine trials is clearly useful to pinpoint potential biomarkers of interest in assessing sensitivity or resistance to MTAs. We focused on patients who experienced an objective response or disease stabilization that lasted longer than 6 months according to RECIST. Whereas an objective response with a single-agent MTA clearly indicates treatment efficacy, using prolonged disease stabilization as a criterion is questionable.^25^ The example of the long-responder patients’ analysis, although bearing the above limitations, highlights the precious information that could be inferred from precision medicine trials’ data analyses. 

Many lessons could definitely be learned using this approach: the possibility to focus on patients’ subpopulation with specific clinical or molecular questions within the same prospective study (in our case, patients experiencing an objective response or prolonged disease stabilization following treatment within the SHIVA01 trial); the accessibility of centralized molecular data obtained using the same techniques and bioinformatics pipelines and thus avoiding multiple sites biases; the identification of potential biomarkers of interest depending on the question asked (in our case, global number of molecular alterations, several alterations in the same pathways, or TP53 mutations) that required additional validation in independent cohorts; and the importance of sharing data with other precision medicine clinical trials to enlarge specific subpopulation or to validate results. 

The SHIVA01 trial did not involve immunotherapy as it started before the immunotherapy era. Tumor mutational burden and microsatellite instability have been suggested as potential biomarkers of efficacy for immune checkpoint inhibitors.^26-28^ It remains to be determined how the incorporation of immunotherapy in SHIVA01 would have affected the results. In addition, it cannot be excluded that these parameters might contribute to a better understanding of the prolonged responses reported here.
